# Protein Lipidation by Palmitoylation and Myristoylation in Cancer

**DOI:** 10.3389/fcell.2021.673647

**Published:** 2021-05-20

**Authors:** Chee Wai Fhu, Azhar Ali

**Affiliations:** Cancer Science Institute of Singapore, National University of Singapore, Singapore

**Keywords:** protein lipidation, palmitoylation, myristoylation, depalmitoylation, metabolism, cancer

## Abstract

Posttranslational modification of proteins with lipid moieties is known as protein lipidation. The attachment of a lipid molecule to proteins endows distinct properties, which affect their hydrophobicity, structural stability, localization, trafficking between membrane compartments, and influences its interaction with effectors. Lipids or lipid metabolites can serve as substrates for lipidation, and the availability of these lipid substrates are tightly regulated by cellular metabolism. Palmitoylation and myristoylation represent the two most common protein lipid modifications, and dysregulation of protein lipidation is strongly linked to various diseases such as metabolic syndromes and cancers. In this review, we present recent developments in our understanding on the roles of palmitoylation and myristoylation, and their significance in modulating cancer metabolism toward cancer initiation and progression.

## Introduction

The hallmarks of cancer are characterized by biological properties, which include continuous proliferation, resistance to apoptosis, metastasis and epithelial mesenchymal transition, sustained angiogenesis, and metabolic reprogramming (Hanahan and Weinberg, 2011). Cancer phenotypes are attributed by the function of oncoproteins affected by posttranslational modifications (PTMs) in tumor cells (Lothrop et al., 2013). PTMs alter subcellular localization, stability, and activity of protein molecules. Multiple classes of PTMs have been identified in mammalian cells including phosphorylation, acetylation, sumoylation, and lipidation (Lothrop et al., 2013; Blanc et al., 2015). Protein lipidation, one of the most important and diverse classes of PTMs, can reversibly or irreversibly attach up to six different lipid types including fatty acids, isoprenoids, sterols, phospholipids, glycosylphosphatidylinositol (GPI) anchors, and lipid-derived electrophiles (LDEs) to proteins. Protein lipidation is hypothesized to be tightly regulated and may partially contribute to the pathogenesis of cancer with dysregulated lipid metabolism. Palmitoylation, myristoylation, and farnesylation are the three major lipidation processes. It is, therefore, important to understand the mechanisms, functions and pathological relevance of protein lipidation, which can ultimately lead to identification of novel therapeutic targets. In this review, we will cover the two most common protein lipidation, palmitoylation and myristoylation, and discuss their roles on cancer initiation and pathogenesis.

## Palmitoylation

Palmitoylation is a dynamic process where it influences protein distribution, localization, accumulation, secretion, stability, and function by altering the protein’s membrane affinity. Through specific methods such as radioactive isotope-labeled lipids or acyl-biotin exchange, early studies have identified hundreds of cancer-related protein palmitoylation (Kang et al., 2008; Yang et al., 2010; Ivaldi et al., 2012; Chen et al., 2018). These proteins can either be mono-palmitoylated or poly-palmitoylated, and the presence of palmitoylated proteins in tumor cells indicate a functional role in cancer. Palmitoylation is categorized into S-palmitoylation or the less frequently occurring O-palmitoylation and N-palmitoylation. O-palmitoylation is the addition of fatty acyl group to serine residues, while N-palmitoylation is the addition of fatty acyl group to the N-terminus. To date, a few oncoproteins are identified to be O- or N-palmitoylated where two well-known examples of O-palmitoylated proteins are Wnt and Histone H4 (Zou et al., 2011; Miranda et al., 2014), while Hedgehog proteins are N-palmitoylated (Pepinsky et al., 1998).

### S-Palmitoylation

**FIGURE 1:**
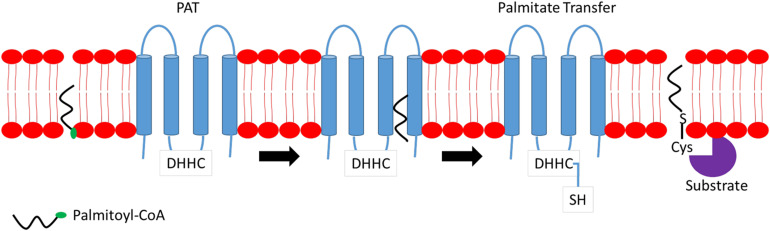
Mechanism of action of palmitoyl S-acyltransferase (PAT). Briefly, Asp–His–His–Cys (DHHC) binds to palmitoyl-coenzyme A (CoA) located at the membrane and transfers a fatty acyl chain to the targeted substrate protein releasing CoA.

S-palmitoylation is the major form of palmitoylation and involves the addition of fatty acyl group, usually palmitic acid, to cysteine residues of protein. In certain circumstances, other fatty acids with varied carbon lengths are utilized (Senyilmaz et al., 2015). Due to the labile nature of thioester bonds, S-palmitoylation is reversible and dynamic, and S-palmitoylated proteins can undergo cycles of palmitoylation and de-palmitoylation within seconds to hours in response to upstream signals. Some proteins are exclusively S-palmitoylated, while others undergo a combination of S-palmitoylation and an additional modification of protein lipidation such as myristoylation or farnesylation. Examples of such proteins include the Src family of kinase, p59fyn, and H-Ras (Cadwallader et al., 1994). Although S-palmitoylation was discovered several decades ago, little is known on the mechanisms and participating players involved in both palmitoylation and de-palmitoylation processes and, more importantly, the recognition of protein sequences on substrate proteins for palmitoylation. S-palmitoylation is catalyzed by a specific class of enzymes called palmitoyl S-acyltransferases (PATs). PATs family comprises 23 distinct members in mammals. Active sites of PATs contain zinc finger Asp–His–His–Cys (DHHC) domain required for palmitic acid transferring activity to substrate protein (Putilina et al., 1999). Most DHHC proteins are localized primarily to the endoplasmic reticulum (ER) and Golgi apparatus. Figure 1 provides a simplified mechanism of action of PAT. Briefly, the Asp–His–His–Cys (DHHC) domain is critical for PAT enzymatic activity where palmitoyl-CoA reacts with the cysteine residue within the DHHC motif, forming an acyl intermediate and subsequent release of coenzyme A (CoA-SH). The fatty acid chain is then transferred directly to a substrate protein. Cysteine residues, which are located close to the DHHC domain, is essential as it anchors two zinc atoms for proper enzyme folding and function; however, it does not play any catalytic role in palmitate transfer (González Montoro et al., 2013).

Despite the high similarity in amino acid sequences between all DHHC enzymes, each individual DHHC member exhibits distinctive differences in catalytic efficiency and fatty acyl preferences. Structural and functional studies have shown a region in each DHHC enzyme that promotes substrate interaction and palmitoylation, thus, suggesting unique substrate-binding preferences guiding palmitoylation of certain proteins. However, the exact consensus palmitoylation sequence remains unknown, and the mechanism(s) as to how each individual DHHC enzyme selects a specific substrate for palmitoylation remains entirely unclear. S-palmitoylation is a highly specific modification process on a given protein, and it occurs on a particular internal cysteine residue. The success of palmitoylation on peripheral proteins though is dependent on DHHC–substrate binding conformation. One such example is the DHHC7-mediated scribble planar cell polarity protein (SCRIB) palmitoylation. DHHC7 palmitoylates SCRIB to promote tumorigenesis where DHHC7 is shown to interact with N-terminal leucine-rich repeat (LRR) domain of SCRIB. Site-directed mutagenesis at amino acid residue 305 from proline to leucine (P305L) at the LRR domain affects its local structure where increased binding is observed between DHHC7 and SCRIB P305L mutant. Furthermore, DHHC7 is unable to transfer palmitate acid to CRIB P305L mutant due to its altered binding conformation (Chen et al., 2016). These observations highlight the importance of correct DHHC–substrate binding conformation for palmitoylation to occur.

Asp–His–His–Cys enzymes exhibit redundancy in substrate binding. However, each DHHC also exhibits stronger binding affinity and palmitoylation efficiency toward specific protein substrates. DHHC enzymes’ substrate selection and binding are affected by three main factors. Subcellular localization is the first factor for palmitoylation to occur, a substrate protein has to be bound to the membrane and interacts with a DHHC enzyme. For certain proteins, this can be accomplished by undergoing an initial lipidation event such as prenylation and myristoylation prior to the palmitoylation process. The initial lipidation event must occur at the amino acid residue positioned in close proximity to the Cys residue(s) to be palmitoylated, thus, rendering help to the protein, which is bound weakly to the membrane and facilitating their anchoring to the DHHC enzyme. Two protein examples on the requirement of pre-lipidation event are RAS C-terminal farnesylation and Src-family N-terminal myristoylation (Willumsen et al., 1984; Hancock et al., 1991; Patwardhan and Resh, 2010). Another factor that regulates DHHC activity and expression is the presence of specific accessory or cofactor proteins. DHHC9 is shown to form a stable complex with GCP16, which helps stabilize DHCC9 in HEK293 cells. In the absence of GCP16, DHHC9 partially undergoes proteolysis. Furthermore, DHHC9 fails to S-acylate H-Ras in the absence of GCP16 suggesting that GCP16 does not only stabilizes DHHC9 but also contributes to DHHC9 S-acylation reaction (Swarthout et al., 2005).

### Depalmitoylation

S-palmitoylation is a dynamic and reversible modification process, and depalmitoylation is carried out by the enzyme cysteine deacylase. This “eraser” enzyme belongs to the family of serine hydrolases. Acyl-protein thioesterase (APT) is the first depalmitoylation enzyme identified. There are two APT paralogs – APT1 and APT2. Both isoforms are ∼64% identical and share a similar structure (Toyoda et al., 1999). However, they exhibit a different localization pattern within the cell. APT2 is a cytosolic protein, whereas APT1 is found in both cytosol and mitochondria (Kathayat et al., 2018). Different cellular distributions may contribute to the functional differences between APT1 and APT2 functions. As cytosolic proteins, APT1 and APT2 do share some common targeted proteins such as H-Ras, growth-associated protein-43 (GAP-43) (Kong et al., 2013), and NMNAT2 (Milde and Coleman, 2014). Until now, the consensus sequences flanking the thioacyl group, which is recognized by APT1/2, has not been reported. APTs do not depalmitoylate substrate proteins indiscriminately, and this is shown by failure to remove the acyl group from acylated caveolin by recombinant APT1, whereas endothelial nitric oxide synthase (eNOS) diacylation is readily noticed (Dietzen et al., 1995; Yeh et al., 1999).

Palmitoyl protein thioesterase 1 (PPT1) exhibits capability to depalmitoylate [^3^H]-palmitate-labeled H-Ras, Gα subunits, and acyl-CoA *in vitro*, with preference to 14–18 carbon lengths (Camp and Hofmann, 1993; Camp et al., 1994). PPT1 is a lysosomal protein and unlikely to play any role in deacylating cytoplasmic proteins (Verkruyse and Hofmann, 1996). PPT2, a lysosomal protein as well, possesses a substrate specificity for palmitoyl-CoA and not palmitoylated proteins. In recent years, a family of mammalians α/β hydrolase domain containing proteins (ABHD), comprising at least 19 members, has been proposed to be depalmitoylation enzymes. Within the ABHD family, ABHD17 hydrolase is the most well-studied protein, and it is divided into three subspecies: ABHD17A, ABHD17B, and ABHD17C, which are broadly expressed in all vertebrates and harbor multiple conserved cysteine residues near their N-termini. ABHD17 has been reported to regulate palmitate turnover on postsynaptic density protein 95 (PSD95) and N-Ras (Lin and Conibear, 2015).

### Functional Interactions Between Palmitoylation and Depalmitoylation Enzymes

**FIGURE 2:**
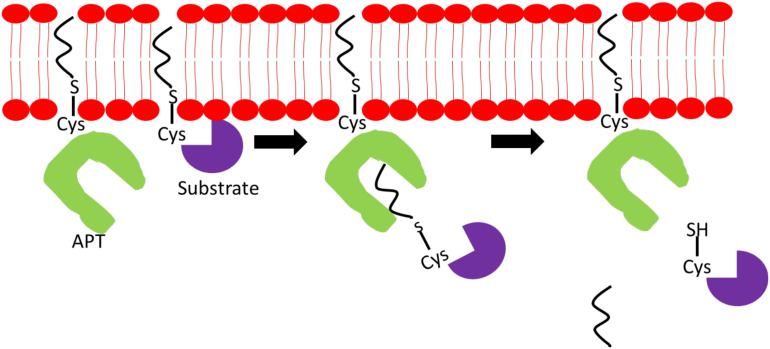
Mechanism of action of acyl-protein thioesterase (APT). APT binds to a specific acylated substrate and cleaves off the fatty acyl chain located on the sulfur atom on the cysteine residue linked via thioester bond.

Due to the continuous cycles of protein palmitoylation and depalmitoylation to maintain optimal cellular functions, PPTs, APTs, and DHHCs have to work cooperatively and coexist in highly complex networks. Palmitoylation of APT1/2 and ABHD-family of thioesterases is critical for their proper localization and function. Palmitoylation of APT1 and APT2, at cysteine-2 residues, facilitates their cytosol-membrane shuttling and cellular membrane anchoring to execute depalmitoylation of Ras and GAP-43 proteins. Adding further complexity to the network, APT1 also regulates APT2 palmitoylation levels but not vice versa (Kong et al., 2013). Figure 2 illustrates a simplified mechanism of APTs’ ability to deacylate substrate proteins. ABHD17A-C harbors multiple conserved cysteine residues at the N-termini, which are essential for S-palmitoylation and membrane anchoring (Martin and Cravatt, 2009). Deletion of N-terminal cysteine-rich domain has no effect on ABHD17 activity suggesting that palmitoylation is essential for its membrane localization but not enzymatic activity. Palmitoylation is shown to affect PPT1 activity, but not its localization, as demonstrated by its palmitoylation by DHHC3 and DHHC7 enzymes. Palmitoylation of mutated PPT1 at C6S did not alter its intracellular location; however, the non-palmitoylated form of PPT1 exhibited a higher depalmitoylation activity (Segal-Salto et al., 2016). Under certain conditions, a single palmitoylation process involves multiple DHHC enzymes and APTs as observed in the complex network surrounding DHHC6. To exert its function, DHHC6 requires palmitoylation by DHHC16 at three cysteine residues in its SH3_2 domain. This acylation is required for the subsequent acylation of its substrates, calnexin and transferrin receptor, at the ER. Although palmitoylated DHHC6 possesses high acylation activity, it also contributes DHHC6 to undergo ER-regulated degradation thus providing a reason as to why DHHC6 needs to be depalmitoylated rapidly by APT2 to maintain its cellular level (Abrami et al., 2017).

## N-Myristoylation

**FIGURE 3:**
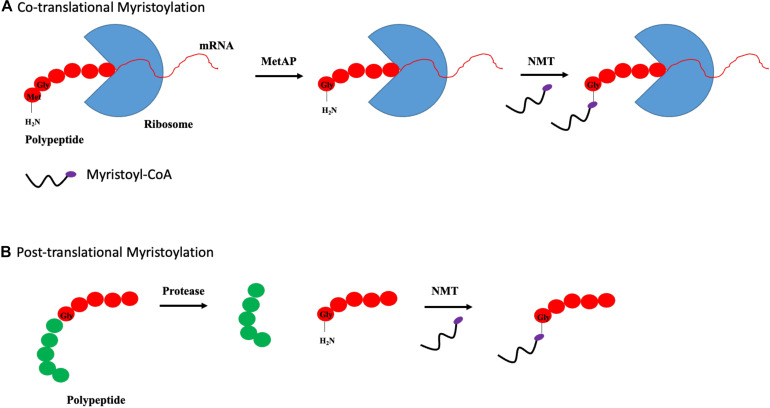
Types of myristoylation. **(A)** Schematic illustration of steps involved in cotranslational myristoylation. During cotranslational myristoylation, the myristoyl group is added to the N-terminal glycine residue following cleavage of the N-terminal methionine residue on the growing polypeptide chain. **(B)** Posttranslational myristoylation normally occurs following caspase cleavage event, resulting in the exposure of internal glycine residue, which allows myristic acid addition.

N-myristoylation is a process involving the covalent attachment of myristate, a 14-carbon saturated fatty acid, to the N-terminal glycine residue of protein (Farazi et al., 2001). The N-myristoylation process is catalyzed by N-myristoyltransferase (NMT), which is a member of the Gcn5-related N-acetyltransferase (GNAT) superfamily. In contrast to palmitoylation, myristoylation is irreversible and can occur co- and posttranslationally. Cotranslational myristoylation occurs when an initiator methionine residue is removed by methionine aminopeptidase, followed by the attachment of NMT-regulated myristate. The entire process occurs more efficiently when a glycine residue is located right after the initiator methionine as shown in Figure 3A (Frottin et al., 2006). Posttranslational myristoylation takes place when the internal glycine residue, within a cryptic myristoylation consensus sequence, is exposed by the action of caspase in apoptotic cells as shown by Figure 3B (Zha et al., 2000). Similar to S-palmitoylation, N-myristoylation is critical for subcellular targeting, protein–protein and protein–membrane interactions, and is required for the activities of certain oncoproteins including Src and ADP-ribosylation factor 1 (ARF1) (Liu et al., 2009; Patwardhan and Resh, 2010). Although protein myristoylation is necessary for membrane binding, it must be augmented with additional downstream modification for enhanced membrane anchoring (Peitzsch and McLaughlin, 1993; Shahinian and Silvius, 1995). A well-known example would be the Src family of kinases, in which Src protein requires both myristoylation and palmitoylation to accurately position itself at the cellular membrane, and myristoylation is shown to be a pre-requisite for palmitoylation to occur (Alland et al., 1994; Koegl et al., 1994). While myristoylation modification is irreversible, acylated proteins can bind reversibly to membrane owing to its weak hydrophobic nature. The half-life of a myristoylated protein bound to membrane is in the order of minutes, in contrast to hours for palmitoylated or double (myristoylated and palmitoylated) modified protein (Martin et al., 2011). It is postulated that the orientation of myristoyl moiety within the protein is highly dynamic. N-myristoylated protein can exist in two conformations where the myristoyl moiety is either sequestered in a hydrophobic pocket within the protein or flipped out and exposed to participate in membrane binding. The transition between these two conformations is regulated by a “myristoyl switch.” “Myristoyl switch” is categorized into three classes: ligand binding, electrostatic, and proteolysis. Recoverin is a calcium-binding protein in retina that regulates the phosphorylation of photoexcited rhodopsin through rhodopsin kinase inhibition. In a calcium-free environment, the myristoyl group is sequestered in a hydrophobic pocket where calcium-binding induces a conformational change within recoverin and releases myristate for membrane binding (Tanaka et al., 1995).

Another factor that influences myristol group-membrane binding is electrostatic charge. Myristoylated alanine-rich c-kinase substrate (MARCKS) protein is a substrate of protein kinase C (PKC) where myristoylated MARCKS is displaced from the membrane upon phosphorylation by PKC. MARCKS phosphorylation, occurring within the basic domain, introduces negative charge to the positively charged region. This reduces interaction between the positively charge myristoylated region of protein and negatively charged acidic phospholipids thus leading to its removal from the membrane (Thelen et al., 1991). The significance of electrostatic charge on membrane-binding Src is demonstrated where myristoylation of Src contributes to its strong affinity binding to plasma membrane. The strength of this interaction is ∼2,500-fold higher when binding occurs on vesicles with membranes containing a physiological ratio of 2:1 phosphatidylcholine (PC)/phosphatidylserine (PS) than the neutral PC bilayer. Src mutants, with basic residues at the amino terminus replaced by neutral asparagine, exhibit a significant reduction in interaction strength (Sigal et al., 1994). The final factor affecting myristoylated protein–membrane interaction is the myristoyl–proteolytic switch. An example to illustrate this modification is the human immunodeficiency virus type I gene (HIV-1) Pr55^gag^. Pr55^gag^ is directed to the plasma membrane by a myristate with basic motif (Zhou et al., 1994). Myristoylation at the N-terminal, together with the highly basic region, is critical to acid phospholipid membrane binding. Pr55^gag^ is cleaved by HIV-1 protease to yield N-terminal cleavage product, P17MA. This cleavage process triggers myristoyl switch where the myristate group is found sequestered in P17MA, thus, rendering low binding affinity to the membrane (Hermida-Matsumoto and Resh, 1999). This feature is highly critical for the infectivity of HIV virus.

### N-Myristoyltransferase

N-myristoylation is catalyzed by the enzyme N-myristoyltransferase (NMT). The mechanism of action by NMT, in *Saccharomyces cerevisiae*, shows that this enzyme performs its catalyst activity via an ordered Bi-bi reaction. Myristoyl-CoA first binds to NMT followed by a peptide substrate and subsequent transfer of myristyl-CoA to N-terminal glycine residue of substrate. CoA molecule and myristoylated protein are then released from NMT at the end of the process (Rudnick et al., 1991). Two human NMT isoforms have been identified, namely, NMT1 and NMT2, and they share ∼77% amino acid sequence similarity (Giang and Cravatt, 1998). Similar to DHHC, both NMTs exhibit distinctive differences in function and demonstrate a certain degree of functional redundancy. This is clearly shown by NMT1 knock-out (NMT^–/–^) mice suggesting that NMT1 is critical for early embryonic development. Intercross of NMT heterozygous mice did not produce any NMT^–/–^ offspring as NMT^–/–^ embryos died between embryonic days 3.5 and 7.5, and heterozygotes offspring were born at a lesser frequency. Furthermore, NMT^–/–^ embryonic stem (ES) cells revealed that, although NMT2 isoform was detected in ES cells, total NMT activity was greatly reduced by ∼95%. These data suggest that NMT2 expression does not compensate for loss of NMT1 during embryogenesis (Yang et al., 2005). NMT1 and NMT2 share redundant and specific effects on protein processing, apoptosis, and cellular proliferation. Specific small interfering RNAs (siRNAs) against each individual NMT isoform can reduce the expressions of both NMT isoforms by at least 90%, respectively. Ablation of NMT1, but not NMT2 expression, can suppress cellular proliferation through reduced Src activation and signaling *in vitro* and *in vivo* models. However, suppression of both NMT1 and NMT2 can induce apoptotic effect on ovarian cancer cell line, SK-OV-3, with NMT2 inhibition showing greater apoptotic effect compared with NMT1 (Ducker et al., 2005).

N-myristoyltransferases possess preferential fatty acyl substrate specificities where 14-carbon chain myristoyl-CoA is the preferred substrate. It is noteworthy to mention that palmitoyl-CoA can also bind to NMT with approximately the same Michaelis constant (K_m_) value as myristoyl-CoA; however, palmitoyl-CoA is not transferred to proteins by NMT. It is intriguing how NMT selects myristoyl-CoA over palmitoyl-CoA, which exhibits similar binding affinity and higher intracellular concentrations (palmitoyl-CoA is ∼5- to 20-fold higher). Structural analyses of various fatty acyl-CoA analogs show that the geometry of enzyme’s acyl-CoA binding site requires the acyl chain of active substrate to acquire a bend confirmation within the C5 vicinity. Furthermore, the distance between C1 and bend is critical for optimal positioning of acyl-CoA for peptide substrate binding through ordered Bi-bi reaction mechanism (Rudnick et al., 1992). Additionally, carbon chain length of fatty acyl-CoA rather than hydrophobicity plays a determinant role in NMT substrate selection (Heuckeroth et al., 1988). Together, these data demonstrate that myristoyl-CoA is the preferred substrate for NMT instead of palmitoyl-CoA.

### Lysine Acylation and Deacylation

Lysine residue was first discovered to be myristoylated on the membrane-bound precursors of cytokine interleukin 1-α (IL-1) and tumor necrosis factor-α (TNF-α) about 30 years ago (Stevenson et al., 1992, 1993). Apart from myristoylation, recent findings revealed that lysine residues can be palmitoylated, and lysine fatty acylation has garnered much attention over the years due to the diverse functions of lysine fatty acylation (Wilson et al., 2011). Until now, protein candidates, which are responsible for lysine fatty acylation or de-acylation are still unknown. There are numerous ongoing studies to identify the players involved in the lysine acylation process. Recently, an unexpected function of NMT1 and NMT2 was reported where they were found to efficiently myristoylate lysine 3 residue on ARF6. This step is essential for ARF6 attachment to the membrane during GTPase cycle. More importantly, this study shows that Sirtuin (Sirt) 2 can remove the myristoyl group from lysine residue on ARF6 suggesting a complex network of NMT/Sirt2-ARF6 regulatory network in GTPase cycle (Kosciuk et al., 2020). Sirtuin belongs to the family of seven NAD-dependent deacetylases that remove the acetyl group from acetylated histone. However, it was reported in *in vitro* assays that deacetylase activity in certain members of the Sirt family is weak, and they are more catalytically efficient toward long-chain peptide substrate (myristoylated) compared with acetylated peptide substrate (Jiang et al., 2013; Feldman et al., 2015).

The physiology significance of Sirt deacylase activity started after the discovery of its involvement in demyristoylating a lysine residue in TNF-α maturation and extracellular secretion. Sirt 6 is shown to hydrolyze the myristoyl group of both lysine 19 (K19) and 20 (K20), which helps in matured TNF-α secretion (Jiang et al., 2013). Replacement of K19 and K20 with arginine affects TNF-α secretion, which is promoted by Sirt 6 deacylation activity. Progressively, the candidates for Sirt deacylase activity are shown to include K-Ras 4a (Jing et al., 2017) and RalB (Spiegelman et al., 2019); the diacylation of these proteins play essential roles in cancer progression.

Interestingly, findings *in vitro* demonstrates reciprocal regulation of deacetylation, and diacylation showed that the addition of long-chain fatty acid such as palmitic and myristoylic acid can stimulate Sirt 6 deacetylated activity on H3K9Ac. A detailed steady-state inhibition analysis revealed that myristoylic acid competes with the myristoylated peptide for the same binding pocket on Sirt 6, which eventually led to a lower demyristoylation activity. Binding of myristoylic acid to Sirt 6 also contributes to conformational changes, thus, allowing efficient deacetylation (Feldman et al., 2013).

## Palmitoylation and N-Myristoylation in Cancer

Palmitoylation and myristoylation are shown to play crucial roles in cancer progression as demonstrated by past studies investigating the roles of DHHCs, APTs, and NMTs in aberrant activation of oncogenic signaling networks such as Src and Ras, among others. The aberrant oncogene signaling confers a more aggressive, and imparts a higher proliferative, capacity of cancer cells. However, the impact of palmitoylation or myristoylation on cancer metabolism such as mitochondrial respiration, glycolysis, and fatty acid oxidation (FAO) are not well documented. Cancer metabolism has now garnered attention since its inclusion as a hallmark of cancer (Hanahan and Weinberg, 2011). Cancer metabolism is highly dynamic and complex, and disruption of an arm of a metabolic process can shift the entire cellular metabolism in an attempt to maintain a new equilibrium for survival. Cancer lipid metabolism is also found to play an essential link between lipid synthesis and lipid consumption by providing the building blocks required for membrane synthesis as well as lipid moieties for protein lipidation. Hence, we will discuss the impact of protein lipidation on cancer metabolism in this review.

### Palmitoylation/Depalmitoylation and Cancer Metabolism

Due to technological advances in research, the list of identified palmitoylated proteins is growing (Table 1). Proteins that can be palmitoylated are shown to span across various categories including proteins involved in cellular metabolic regulation particularly mitochondrial proteins. Hence, factors that can influence metabolic protein palmitoylation are postulated to affect cancer cell respiration as well. Cellular metabolism is tightly dependent on various variables such as nutrient uptake, morphology of mitochondria, and metabolic enzyme activities. Hepatic cell plays an important role in metabolizing lipids in the body. Palmitoyltransferases DHHC4 and DHHC5 regulate fatty acid uptake by palmitoylating and targeting CD36 to the plasma membrane. DHHC4 is shown localized at the Golgi apparatus, while DHHC5 can be found at the plasma membrane. Depletion of either DHH4 or 5 disrupts fatty acid uptake capability of adipose tissues. Furthermore, both DHHC4 knock-out or adipose-specific DHHC5 knock-out mice exhibit reduced fatty acid uptake. DHHC4 and DHHC5 isoforms are not functionally redundant and do not share a similar localization pattern within the cell (Wang et al., 2019). Prior progressing into liver cancer, non-alcoholic steatohepatitis (NASH) features abnormal lipid metabolism with palmitoylated CD36 upregulation. CD36 functions as a fatty acid transporter, and its expression is significantly enhanced at cellular plasma membrane in NASH samples. In the NASH mouse model, enhanced expression of membranous CD36 is linked to increased palmitoylation on CD36, which, in turn, helps CD36 trafficking and anchoring to the cellular membrane. Further validation utilizing a liver cancer cell line, HepG2, cultured under conditions with exogenous palmitic acid, shows upregulation of both CD36 mRNA expression and protein palmitoylation. CD36 palmitoylation inhibition protects mice from developing NASH. Palmitoylated CD36 enhances long-chain fatty acid binding and uptake but lowers FAO through AMPK pathway resulting in lipid accumulation in liver cancer cells. The data clearly shows that palmitoylated CD36 plays an important role in the development of NASH (Zhao et al., 2018).

**TABLE 1:** Summary of palmitoylation and depalmitoylation involvement in cancer metabolism.

Target protein	Modification	Enzymes	Consequences	References
CD36	Palmitoylation	DHHC4 and DHHC5	Increased fatty acid uptake and fatty acid oxidation	Zhao et al., 2018; Wang et al., 2019
Erα	Palmitoylation	DHHC7 and DHHC21	Increased glucose uptake	Pedram et al., 2012; Garrido et al., 2013
GLUT4	Palmitoylation	DHHC7	Increased glucose uptake	Ren et al., 2015
KRAS4A	Depalmitoylation	Unknown	Increased glycolytic flux	Amendola et al., 2019
TMX-1	Palmitoylation	Unknown	Increased mitochondrial respiration and ATP production	Raturi et al., 2016
CKAP4	Palmitoylation	DHHC2	Increased basal mitochondrial respiration and maximal respiratory activity	Zhang et al., 2008; Harada et al., 2020
DRP-1	Palmitoylation	DHHC13	Increased oxidative phosphorylation	Napoli et al., 2017
Mitochondrial EGFR	Palmitoylation	Unknown	Increased mitochondrial fusion	Bollu et al., 2014
PRDX5	Depalmitoylation	ABHD10	Increased mitochondrial redox buffering capacity	Cao et al., 2019
Malonyl CoA-acyl carrier protein transacylase and Catenin delta-a	Palmitoylation	DHHC13	Decreased mitochondrial function and increased oxidative stress	Shen et al., 2017

Apart from fatty acid uptake and oxidation, palmitoylation influences glucose uptake as demonstrated in estrogen receptor (ER)-positive breast cancer. Both DHHC7 and 21 can palmitoylate ERα at cysteine 451 (Pedram et al., 2012). Using an ER-positive breast cancer cell line, MCF-7, the activation of ERα receptors enhance glucose uptake through GLUT4 membrane translocation to fulfill the energy demand of highly proliferative tumor cells. Stimulation of ERα by 17β-estradiol promotes ERα membrane translocation and its concomitant depalmitoylation, and allows its dissociation from the plasma membrane to interact with proximal kinases, PI3K/Akt. Activated ERα interacts with p85α, a key member of the insulin-signaling cascade, and forms a complex capable of inducing Akt activation through Ser473 phosphorylation. This eventually leads to GLUT4 membrane translocation and enhances cellular glucose uptake that can be inhibited using PI3K/Akt-specific inhibitor, LY294002 (Garrido et al., 2013). Interestingly, direct GLUT4 palmitoylation, at Cys223, induces GLUT4 membrane translocation and improves glucose uptake in 3T3 cell line (Ren et al., 2015). DHHC7 has been subsequently identified to be the PAT responsible for GLUT4 palmitoylation (Du et al., 2017). Palmitoylation also regulates hexokinase 1 (HK1) activity in glycolysis. Depalmitoylation of KRAS4A, at cysteine 180 residue, is crucial for HK1 association at the outer mitochondria membrane (OMM). Palmitoylation-deficient KRAS4A mutant and 2-BP treatment reduce KRAS4A-HK1 association at the OMM. KRAS4A-HK1 association ameliorates the inhibitory effect of 2-deoxyglucose (2-DG) on HK-1 suggesting a functional role of KRAS4A-HK1 association. Binding of KRAS4A to HK1 is shown to boost HK1 activity, induces higher glycolytic flux with both glucose consumption, and basal extracellular acidification rates are elevated significantly (Amendola et al., 2019).

Cancer metabolism is highly dependent on the physiological state of the mitochondria. The mitochondrion and endoplasmic reticulum (ER) are connected through sites called mitochondrial-associated membrane (MAM). MAM is important for the regulation of Ca^2+^ flux between ER and mitochondrion to achieve optimal cellular function and mitochondrial ATP production. ER-localized thioredoxin-related transmembrane protein (TMX) is shown to localize at MAM sites in a palmitoylation-dependent manner. Comparisons between wild type and mutated TMX, with altered putative palmitoylated sites, revealed that altered dual palmitoylation sequence reduces mutant TMX level in the MAM from >50% to <20%. Furthermore, inhibition of TMX palmitoylation using 2-bromopalmitate (2-BP) diminishes TMX levels at MAM sites (Lynes et al., 2012). These observations are supported in a separate study where palmitoylated TMX-1 is observed to interact with SERCA2b in a calnexin-dependent manner to regulate Ca^2+^ flux between the ER and the mitochondria. Modification of palmitoylated sites from cysteine to alanine on TMX-1 impairs its MAM localization and ability to interact with SERCA2b. Furthermore, suppression of TMX-1 expression induces high retention of Ca^2+^ in the ER and low Ca^2+^ flux to the mitochondria, which indirectly affects its metabolism through ER. Respiratory capacity of TMX-1-knocked down Hela cells is lowered by 50% when compared with scrambled. Furthermore, mitochondria capacity of the melanoma cell line, A375, is shown to be elevated by 20% when TMX-1 levels are overexpressed in these cells. TMX-1 knockdown in Hela and A375 cells is shown to significantly reduce ATP production when compared with the respective controls (Raturi et al., 2016).

Cytoskeleton-associated protein 4 (CKAP4), which is localized at MAM sites, is reported to exert its role in cellular respiration and requires palmitoylation to achieve optimal function. CKAP4 is a known substrate of DHHC2 (Zhang et al., 2008). Structural analyses of mitochondria in CKAP4 KO cells revealed significant fragmentation and attenuated mitochondrial network when compared with tubular mitochondrial reticulum in control cells. CKAP4 KO cells also exhibit increased mitochondrial number but smaller in mitochondrial sizes compared with the control. Furthermore, CKAP4 KO cells possess lower basal oxygen consumption and maximal respiration capacity, and the levels of oxidative phosphorylation-related proteins remained unchanged. CKAP4 has been proposed to regulate mitochondrial function by forming a functional complex with voltage-dependent anion-selective channel protein 2 (VDAC2). Formation of this complex, however, requires palmitoylation of CKAP4. A palmitoylation-deficient variant of CKAP4 and CKAP4^C100S^ is shown to be incapable of binding to VDAC2 and disrupts mitochondrial function. Expression of CKAP4^WT^ or CKAP^C100S^ in CKAP4 KO cells, which show abnormal mitochondrial structure and oxidative phosphorylation, can be rescued by CKAP4^WT^ but not CKAP^C100S^. These evidence highlight the importance of palmitoylation for proper CKAP4 function. Furthermore, SCID mice carrying a tumor with CKAP KO and CKAP^C100S^ showed a significantly smaller tumor volume and mass due to reduced mitochondria size and function (Harada et al., 2020).

Palmitoylation can directly affect mitochondria protein function. 2-BP treatment is shown to shift the balance of mitochondrial fusion and fission by influencing dynamin-related protein 1 (Drp-1) and optic atrophy 1 (OPA1) levels. 2-BP relieves bone cancer pain through disruption of mitochondrial dynamics together with reduced production of proapoptotic factors and proinflammatory cytokines. In a bone cancer pain rat model (BCP) inoculated with MRMT-1 rat mammary gland carcinoma cells, proteins from spinal cord extracts showed significant upregulation of Drp-1 expression in BCP rats compared with sham, and 2-BP treatment attenuated Drp-1 levels. Conversely, OPA-1 expression is downregulated extensively in the BCP group compared with sham, and 2-BP treatment restores OPA-1 expression (Meng et al., 2019). The impact of 2-BP on Drp-1 is further shown where DHHC13 palmitoylates Drp-1 and affects its mitochondrial localization and activity (Napoli et al., 2017). *De novo* synthesis of palmitate by fatty acid synthase (FASN) is reported to contribute to mitochondrial epidermal growth factor receptor (mtEGFR) palmitoylation in mtEGFR-positive prostate and breast cancer cell lines. EGF induces *de novo* palmitate synthesis, and excessive palmitate is shown to assist mtEGFR palmitoylation, which improves its activity by enhancing phosphorylation. Activated mtEGFR promotes mitochondrial fusion by upregulating OPA-1 and prohibitin-2 (PHB-2) expressions where PHB-2 is required to protect OPA-1 from proteolysis. Palmitoylated sites on the mtEGFR have been identified to be cysteines 781, 797, 1,058, and 1,146, and through site-directed mutagenesis, C797 is found critical for both palmitoylation and phosphorylation of mtEGFR (Bollu et al., 2014).

Additionally, a new role of alpha/beta hydrolase domain-containing 10 (ABHD10) in the mitochondria has been proposed. Mitochondrial-specific pan-APT inhibitor is shown to specifically inhibit ABHD10 and affects mitochondrial redox buffering capacity. Subsequent interrogation showed that ABHD10 is a novel mitochondrial APT candidate and possesses mitochondrial S-deacylase activity *in vitro*. Perturbation of ABHD10 expression, in the form of ABHD10 overexpression, upregulates mitochondrial buffering capacity, or hydrogen peroxide lowers this capacity. Peroxiredoxin-5 (PRDX5) is shown to be the downstream target of ABDH10 where cysteine residues at position 100 of PRDX5 are identified to be sites of depalmitoylation and plays a crucial part for its activity (Cao et al., 2019).

### Myristoylation and Cancer Metabolism

Compared with palmitoylation, the available information on myristoylation and cancer respiration is scarce (Table 2). A direct relationship between myristoylation and mitochondrial respiration has yet to be established. However, a possible functional link between myristoylation and mitochondria has been reported where myristoylation is shown to affect AMP-activated protein kinase (AMPK) signaling and participates in mitochondrial surveillance in lung and breast cancer cell lines. When mitochondria are damaged under stress or induced by mitochondrial depolarizing agent, carbonyl cyanide 3-chlorophenylhydrazone (CCCP), an autophagy-related event termed mitophagy is initiated to recycle damaged mitochondria. AMPK is required in both autophagy and mitophagy processes. AMPK is physically shown to associate with damaged mitochondria where upon CCCP treatment, the total and T172-phosphorylated AMPK are upregulated in mitochondrial fractions. Concomitantly, recruited AMPK initiates mitophagy process through interaction with ATG16 complexes. AMPK kinase activity is required to accelerate the recruitment of ATG complex following CCCP-induced mitochondria damage. Localization of AMPK is found to be entirely dependent on myristoylation of the β-subunit of AMPK by NMT1. Following CCCP treatment, AMPK is completely abrogated from the mitochondria after the introduction of N-myristoylated-deficient mutant, AMPKβ1G2A-GFP, into cells. The importance of N-myristoylation on AMPK mitochondria localization is confirmed when treatment of cells with myristoylation inhibitor, 2-hydroxymyristic acid (2-HA), suppresses AMPKβ myristoylation and affects its localization at the mitochondria membrane. These observations highlight an important role of myristoylated AMPKβ subunit for proper mitochondria localization and mitophagy process but not for non-selective autophagy (Liang et al., 2015).

**TABLE 2:** Summary of myristoylation involvement in cancer metabolism.

Protein	Modification agent	Consequences	References
AMPK	NMT1	Increased mitophagy	Liang et al., 2015
AMPK	Unknown	Increased fatty acid oxidation	Zhang et al., 2021
SAMM50, MIC19, TOMM40, and MIC25	Unknown	Maintain mitochondrial structure	Utsumi et al., 2018
Akt	Unknown	Increased aerobic glycolysis and reduced fatty acid oxidation	Elstrom et al., 2004; Buzzai et al., 2005; Deberardinis et al., 2006
LAMTOR1	NMT1	Reduced lysosomal degradation	Chen et al., 2020

A link between metabolic reprograming and metastasis through myristoylated AMPK has been recently reported where two isogenic cell lines, one highly metastatic (HM) and the other non-metastatic (NM), were characterized for proteomic and metabolic profiles. Lipid reprograming were observed in HM cells, and expression of ACSL-1 was significantly elevated compared with NM cells. *In vitro* and *in vivo* data suggest that enhanced expression of ACSL-1 contributes to a higher rate of proliferation and confers more tumorigenic properties. Furthermore, higher levels of ACSL-1 seen in HM cells induce a shift in lipidomic profiles through upregulating myristoylic acid production, which strongly associates with cancer progression and metastasis. Excessive amounts of myristoylic acid boost myristoylation process on AMPK leading to AMPK pathway activation and concomitantly upregulates expression of FAO-related ACADM, ACADVL, and HAHDA. To confirm that AMPK myristoylation by ACSL-1 is critical in FAO process regulation, an AMPK inhibitor, dorsomorphin, was utilized, and suppression of AMPK pathway activation failed to promote FAO protein expression even in ACSL-1-overexpressing cells (Zhang et al., 2021).

Myristoylation is critical for proper membrane localization. The same observation also applies to mitochondrial proteins. Myristoylation is detected on four mitochondrial proteins, SAMM50, TOMM40, MIC19, and MIC25, through *in vitro* and *in vivo* metabolic labeling experiments. Myristoylation of these mitochondrial proteins is important for mitochondrial membrane localization and functions as components within the mitochondrial intermembrane space-bridging complex playing a critical role in the maintenance of the mitochondria structure and function. N-myristoylation is shown to play a crucial role in mitochondrial membrane targeting for SAMM50 and MIC19 but not TOMM40 and MIC25. Data from immunoprecipitation assays suggest that MIC19 is a major N-myristoylated binding partner of SAMM50 and that the N-myristoylation of MIC19 is essential for interaction with SAMM50 (Utsumi et al., 2018).

Myristoylation of Akt is critical for its plasma membrane anchoring and confers a higher basal kinase activity. Constitutively active Akt is important to stimulate aerobic glycolysis in cancer cells. Myristoylated Akt is often found overexpressed in leukemia cell lines, and this overexpression is driven by doxycycline. Akt myristoylation can also enhance aerobic glycolysis without affecting oxidative phosphorylation. Excessive amounts of glycolytic end-products are converted to lactate and secreted. These observations suggest that Akt activity contributes to the differences in glucose metabolism rates and glucose dependency for survival. Furthermore, PI3K inhibition, which is upstream of Akt, with LY294002, reduces Akt phosphorylation, glucose consumption rate, and lactate production (Elstrom et al., 2004). Other than glycolysis, Akt myristoylation affects fatty acid metabolism in glioblastoma cells. Glioblastoma cell line, LN18, contains constitutively active Akt and is highly dependent on glucose for survival, while LN229, another glioblastoma cell line, lacks active Akt and exhibits low glucose dependence. Under glucose withdrawal condition, LN18 cell viability is greatly reduced, while LN229 cell survival remains unaffected. Forced myristoylated Akt expression in LN229 (LN229 + myr Akt) induces a metabolic shift, in which cellular viability becomes highly dependent on glucose, and glucose withdrawal greatly reduces LN229 + myr Akt cell viability. Akt activation can suppress the expression of phosphorylated AMPK at T172. Expression of phosphorylated AMPK is crucial to shift the metabolic substrate preference from glucose to fatty acid, and this allows cells to survive under glucose-deprived conditions. Furthermore, LN229 control cells exhibit higher FAO and lower rate of fatty acid synthesis when glucose is removed. However, LN229 + myr Akt cells demonstrate a reverse phenotype where treatment with an AMPK activator, 5-aminoimidazole-4-carboxamide-1-β-D-ribofuranoside (AICAR), improves FAO rate and cell survival rate under a glucose-depleted condition (Buzzai et al., 2005). The suppression of FAO by Akt activation has also been suggested to be a result of carnitine palmitoyltransferase 1A (CPT1A) downregulation (Deberardinis et al., 2006).

Lysosomes are complex cellular organelles involved in modulating cancer cell metabolic homeostasis. NMT1 is necessary for lysosomal degradation and mTORC1 activation in cancer cells. Knock-down of NMT1 in H460 (non-small cell lung carcinoma) and H1792 (lung adenocarcinoma) cells, without affecting the NMT2 expression, is shown to significantly reduce the autophagy flux. Furthermore, decreased NMT1 expression led to reduction in global myristoylation in tumor cells, and this contributed to weakened lysosomal degradation, accumulation of late endosomes/lysosomes, and simultaneous mTORC1 dissociation from lysosome surface leading to attenuated mTORC1 activation. Regulation of lysosomal functions by NMT1 is shown to be mediated through lysosomal adaptor, LAMTOR1. Upon NMT1 knock-down, LAMTOR1 myristoylation is downregulated significantly and failed to position itself at the lysosomal membrane, thus, disrupting mTORC1 activation. All these eventually led to slower cancer cell proliferation (Chen et al., 2020).

## Targeting Protein Lipidation in Cancer

Protein lipidation plays an essential role in cancer progression and initiation, which renders it an attractive cancer therapeutic target. However, the development of anti-lipidation drugs (Table 3) face huge hurdles as illustrated by farnesylation inhibitors, which have shown strong *in vitro* antitumor activity but limited clinical success (Berndt et al., 2011). Nevertheless, several lines of evidence provide strong rationale for targeting PATs as a potential therapeutic avenue. Presently, there are no potent and specific inhibitors against DHHC proteins. 2-Bromopalmitate (2-BP), a broad-spectrum palmitoylation inhibitor, is widely used to validate the anticancer effects of DHHC preclinically. Although it is also referred to as “specific” PAT inhibitor, extensive studies have suggested otherwise. 2-BP treatment has been observed to non-selectively inactivate several types of membrane-bound enzymes including several lipid metabolism-related enzymes including triacylglycerol biosynthesis, fatty acid Co-A ligase, and glycerol-3-phosphate acyltransferase (Coleman et al., 1992) as well as deacylating enzymes, APT1 and APT2 (Pedro et al., 2013). These attributes render 2-BP to be an unreliable lead compound for further development.

**TABLE 3:** Summary of PAT, APT, and NMT inhibitors.

Inhibitors	Blocking target	Structure	Developmental stage	References
2-BP	General depalmitoylation agent	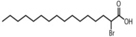	*In vitro* study	Davda et al., 2013
Cerulenin	General depalmitoylation agent	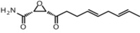	*In vitro* and *in vivo* study	Jochen et al., 1995; Lawrence et al., 1999
Compound V	Reversible inhibitor of palmitoylation and myristoylation	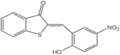	*In vitro* study	Jennings et al., 2009
Tunicamycin	General depalmitoylation agent	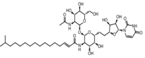	*In vitro* study	Patterson and Skene, 1995; Hurley et al., 2000
ML211	ABHD11	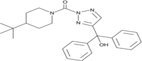	*In vitro* study	Adibekian et al., 2010
ML348	APT1, IC_50_ = 0.21 μM	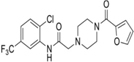	*In vitro* and *in vivo* study	Adibekian et al., 2012; Vujic et al., 2016; Hernandez et al., 2017
ML349	APT2, IC_50_ = 0.144 μM	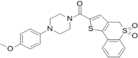	*In vitro* and *in vivo* study	Vujic et al., 2016; Hernandez et al., 2017
Palmostatin B	APT1 and APT2, IC_50_ = 0.67 μM (Ras depalmitoylation)	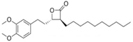	*In vitro* study	Coleman et al., 1992; Hedberg et al., 2011; Pedro et al., 2013; Vujic et al., 2016
Palmostatin M	APT1 and APT2, IC_50_ = 2.5 nM for APT1 and IC_50_ = 19.6 nM for APT2	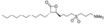	*In vitro* study	Hedberg et al., 2011; Rusch et al., 2011
Mitochondrial pan-APT inhibitor, MitoFP	ABHD10	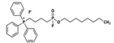	*In vitro study*	Cao et al., 2019
2-Hydroxymyristic acid	NMT, inhibitory activity observed from 100 μM to 1 mM	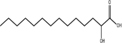	*In vitro* study	Paige et al., 1989, 1990
D-NMAPDD	NMT1, IC_50_ = 77.6 μM	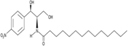	*In vitro* and *in vivo* study	Kim et al., 2017
Tris–DBA palladium	NMT1	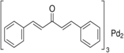	*In vitro* and *in vivo* study	Bhandarkar et al., 2008
IMP-366	NMT1 and NMT2	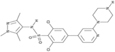	*In vitro* and *in vivo* study	Frearson et al., 2010
IMP-1088	NMT1 and NMT2	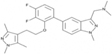	*In vitro* study	Mousnier et al., 2018
PCLX-001	NMT1 and NMT2	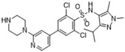	*In vitro*, *in vivo*, and phase I clinical trial	Davda et al., 2013; Randeep et al., 2020; Mackey et al., 2021

The delay in the development of specific DHHC inhibitors lies on the mechanism of action and high similarity protein sequences as well as structures shared among members of the DHHC family. A large number of proteins can be palmitoylated by more than one member of DHHC enzymes. This functional redundancy suggests that specific inhibitors of individual DHHC enzymes may not be clinically useful as they may target multiple DHHC family members. This is similar to the pharmacology of the pan-kinase inhibitor, lapatinib, which inhibits epidermal growth factor receptor and Her2/neu receptor simultaneously. A possible solution to this problem would be to develop pan-DHHC inhibitors that can block the function of several palmitoylation enzymes of a single target. Another way to overcome the limitation is to design specific small molecules targeting specific binding domains of DHHC enzymes. Such an example is the essential ankyrin repeat domains of DHHC13 and DHHC17 for substrate protein to interact and palmitoylate. An alternative method of targeting DHHC enzymes is to promote an irreversible covalent modification of individual cysteine residue with palmitate by targeting protein structure essential for DHHC binding. An example would be the development of covalent inhibitors targeting the transcriptional enhanced associate domain (TEAD) transcription factor. Kojic acid analogs are shown to covalently target the cysteine residues at the central pocket of TEAD. This covalent binding prevents interaction between TEAD with coactivator yes-associated protein (YAP) and reduces TEAD target gene expression (Karatas et al., 2020).

In certain cases, targeting protein depalmitoylation would be more efficient than palmitoylation inhibition. APTs are generally more druggable, and inhibition of APT enzymes can suppress tumor formation. An effort to identify novel APT inhibitors has been based on use of weight loss drug Orlistat, a prototypical serine hydrolase inhibitor, after the discovery that APTs share structural homology with gastric lipase. Structural analysis of Orlistat reveals the presence of an electrophilic β-lactone moiety that covalently inactivates certain serine hydrolases (Hadváry et al., 1991). Modification of β-lactone scaffold has led to the discovery of palmostatin B, the first compound capable of preventing Ras depalmitoylation (Dekker et al., 2010). Further optimization and modification to the chemical structure of palmostatin B led to the development of a better APT inhibitor, palmostatin M. Both inhibitors bind covalently to the serine residue at the active site, thus, blocking the recognition by APTs. Pharmacological analyses of palmostatin B and palmostatin M show that these inhibitors are effective against both APT1 and APT2 without any selective preferences. Palmostatin B and palmostatin M were used to inhibit depalmitoylation of H-Ras (Hedberg et al., 2011). A new class of APT1 and APT2 inhibitors were identified recently, and these potent and non-toxic APT1/2 inhibitors were derived from boronic acid. These compounds were discovered through extensive library screening using chemical arrays, and their effectiveness were later confirmed on Ras protein depalmitoylation *in vitro*. These inhibitors act by competing with APT1/2 at binding sites on the substrate protein. The advantages of these inhibitors are that they exhibit APT isoform specificity and possess slower binding-off rates compared with palmostatin inhibitors (Zimmermann et al., 2013). Depalmitoylation inhibitors could be beneficial for anticancer treatment as most of the oncogene activity or function requires palmitoylation modification for proper localization at the cellular membrane. Furthermore, depalmitoylation inhibitors may be useful to target palmitoylation-induced cancer metabolism reprogramming shown by examples presented utilizing 2-BP. Future studies are required to fully investigate the beneficial effects of depalmitoylation inhibitors in targeting cancer progression or more specifically in targeting cancer metabolism.

N-myristoyltransferase expression and activity are found upregulated in cancers including colorectal (Magnuson et al., 1995), gallbladder (Rajala et al., 2000), and brain (Lu et al., 2005). These observations suggest that NMT expression can be used as biomarkers and a possible cancer therapeutic target. Until now, NMT inhibitors have been used extensively as antifungal agents; however, NMT as a target in cancer is still in the infancy stage. Over the years, several compounds such as 2-hydroxymyristic acid, D-NMAPDD, and Tris–DBA–palladium (Tris–DBA) complex have been utilized as NMT inhibitors in numerous studies. However, these inhibitors are deemed not to be good NMT inhibitors due to several pitfalls. 2-Hydroxymyristic acid, an NMT inhibitor, does not show inhibitory activity at concentrations below 100 μM. Reduced N-myristoylation is only observed when 2-hydroxymyristic acid is applied at concentrations between 100 μM and 1 mM. However, usage at such high concentrations is not favorable due to its precipitation in the culture medium and inducing cell death. Moreover, millimolar concentration is also likely to cause off-target toxicity effects as demonstrated on porcine alveolar macrophages (Du et al., 2010). The application of D-NMAPDD is controversial as it was initially developed as an inhibitor for lysosomal acid ceramidase (Bielawska et al., 1996). D-NMAPDD exhibits NMT1 activity inhibition and reduces Src kinase myristoylation at an IC_50_ of 77.6 μM (Kim et al., 2017). However, this observation was not reproducible in a later study by Kallemeijn et al. (2019). Moreover, D-NMAPDD at 30 μM induces a significant cytotoxic effect within 24 h of exposure by a mechanism unrelated to NMT inhibition as shown by losses of caspase 3 activation and metabolic activity (Kallemeijn et al., 2019). The Tris–DBA palladium complex has been proposed to be an NMT1 inhibitor and inhibits growth of melanoma *in vitro* and *in vivo.* Furthermore, the same complex also shows promising anticancer activity against multiple myeloma (de la Puente et al., 2016), leukemia B cells (Kay et al., 2016), and prostate cancer (Díaz et al., 2016). However, the IC_50_ of the Tris–DBA palladium complex is reported to be 4.2 μM similar to the precipitation concentration in previous findings. It is suggested that the inhibitory effect imparted by the Tris–DBA palladium complex is due to precipitation effect rather than specific interactions. Using similar experimental settings, Kallemeijn et al. (2019) failed to reproduce the NMT1 inhibitory effect of the Tris–DBA palladium complex on A375, a melanoma cell line, as shown by Bhandarkar et al. (2008) in an earlier study. Downregulation of myristoylated protein can be partly explained by the overall reduction in protein production identified through chemical proteomics.

Two small molecule inhibitors, IMP-366 and IMP-1088, were recently developed NMT inhibitors and shown to possess superior specificity. The binding mode of these inhibitors is supported by X-ray co-crystal structural analyses of human NMT1 and NMT2, which demonstrate high specificity of these inhibitors on NMTs. Later on, an improved version of IMP-366 was developed, named PCLX-001, which possessed anticancer activity preclinically. PCLX-001 is reported to possess a greater inhibitory effect in hematological malignancies but less so in solid tumor cell lines. The proposed mechanism of PCLX-001 on tumors is apoptotic cell death through the attenuation of Src myristoylation, basal Src levels, and downstream survival signaling of B-cell receptor (Beauchamp et al., 2020). Other than B-cell lymphoma, PCLX-001 has been reported to effectively kill several breast cancer cell line subtypes ranging from breast carcinoma, ductal carcinoma, and breast adenocarcinoma in both *in vitro* and *in vivo* models (Mackey et al., 2021). The encouraging preclinical data has prompted an open label phase I clinical trial in several Canadian Cancer Centres to establish the antitumor effect maximum tolerable dose of PCLX-001 in B-cell lymphoma and advanced solid tumors. Data obtained from these phase I trials will then be utilized to establish the recommended dose for phase II trial and to evaluate the safety pharmacokinetics of PCLX-001 later on (Randeep et al., 2020).

## Conclusion

In recent years, technological advances have provided better understanding of protein palmitoylation and myristoylation processes. Scientists and researchers are currently exploring the possibility of targeting protein lipidation as a paradigm for cancer therapy; however, there are still some unanswered questions that requires attention. First, apart from DHHC enzymes, it is unclear if there are other enzymes capable of palmitoylating proteins. DHHC enzymes are membrane bound and localized in the ER, Golgi, and plasma membrane. However, there are other non-membrane-bound palmitoylated proteins roaming freely in cells. The recent discovery of LPCAT1, as a palmitoylating agent for histone K4, unveils the possibility of proteins functioning as non-conventional palmitoylation enzymes. Second, the degree of protein palmitoylation of a protein substrate is shown to be highly dependent on the expression and stability of DHHC enzymes as well as APTs, thus, adding further complexity of DHHC enzymes and APT network on palmitoylation. An example would be the DHHC16–DHHC6–APT2 network observed in ER protein palmitoylation. This highlights that DHHC and APT are connected to maintain optimal cellular functions. Understanding the role of each player in this network can help explore and identify possible APTs as a target when a specific inhibitor against the corresponding DHHC is unavailable. Myristoylation process, in general, is less complicated compared with palmitoylation. The greatest concern in targeting myristoylation will be technical challenges that prevents identification of myristoylated proteins in tumor cells. Improved structural analyses and computational approaches are, thus, paramount to accurately identify the three-dimensional structure of myristoylated proteins or NMT for inhibitor development. Furthermore, thorough studies on its efficacy and cytotoxic effect are greatly warranted when exploring the possibility of using NMT inhibitor either as a single or combination therapies against cancers.

## Author Contributions

CF and AA conceptualized the study and wrote and edited the manuscript. AA acquired the funding. Both authors contributed to the article and approved the submitted version.

## Conflict of Interest

The authors declare that the research was conducted in the absence of any commercial or financial relationships that could be construed as a potential conflict of interest.
